# The SR-B1 Receptor as a Potential Target for Treating Glioblastoma

**DOI:** 10.1155/2019/1805841

**Published:** 2019-06-03

**Authors:** Ethan Berney, Nirupama Sabnis, Marlyn Panchoo, Sangram Raut, Rob Dickerman, Andras G. Lacko

**Affiliations:** ^1^Lipoprotein Drug Delivery Laboratory, Department of Physiology/Anatomy, USA; ^2^Neurosurgery, Presbyterian Hospital, Plano, TX, USA; ^3^Department of Pediatrics, University of North Texas Health Science Center, Fort Worth, TX, USA

## Abstract

**Purpose:**

The goal of these studies was to provide proof of concept for a novel targeted therapy for* Glioblastoma Multiforme (GBM). Methods*. These studies involve the evaluation of reconstituted high density lipoprotein (rHDL) nanoparticles (NPs) as delivery agents for the drug, mammalian Target of Rapamycin (mTOR) inhibitor Everolimus (EVR) to GBM cells. Cytotoxicity studies and assessment of downstream effects, including apoptosis, migration, and cell cycle events, were probed, in relation to the expression of scavenger receptor B type 1 (SR-B1) by GBM cells.

**Results:**

Findings from cytotoxicity studies indicate that the rHDL/EVR formulation was 185 times more potent than free EVR against high SR-B1 expressing cell line (LN 229). Cell cycle analysis revealed that rHDL/EVR treated LN229 cells had a 5.8 times higher apoptotic cell population than those treated with EVR. The sensitivity of GBM cells to EVR treatment was strongly correlated with SR-B1 expression.

**Conclusions:**

These studies present strong proof of concept regarding the efficacy of delivering EVR and likely other agents, via a biocompatible transport system, targeted to the SR-B1 receptor that is upregulated in most cancers, including GBM. Targeting the SR-B1 receptor could thus lead to effective personalized therapy of GBM.

## 1. Introduction

Glioblastoma Multiforme (GBM) is a devastating disease with a very poor prognosis, as the survival of patients with GBM rarely extends beyond 3 years from the time of diagnosis [1–3]. Despite intensive research and new approaches to treatment, only limited improvements in patient outcomes have been achieved [4, 5]. New approaches, perhaps involving nanotechnology, are thus urgently needed to improve the survival and the quality of life for GBM patients.

GBM tumors undergo extensive metabolic reprogramming during their development, with epigenetic modifications that are also impacted by the tumor environment [6]. Postoperative hypoxia is likely to facilitate diffuse and invasive tumor growth [7] in addition to enhancing the expression of the scavenger receptor type B1 (SR-B1) [8]. Thus, targeting GBM with a high density lipoprotein (HDL) type drug transporter may be effective against the invasion of GBM tumors, facilitated by the SR-B1 receptor [9].

While nearly 165,000 publications listed in PubMed contain the keyword “nanoparticles” (NPs), the efficiency of payload delivery to oncogenic tissues on the average is only 0.7% [10]. This finding is still perceived as a major impediment to the development of clinical brain barrier application of nanotechnology. In addition, drug resistance to chemotherapy and drug delivery across the blood brain barrier (BBB) are major obstacles to the effective treatment of GBM [1]. Several therapeutic nanocarriers have been for the treatment of GBM including targeted nanoparticles to study the tumor microenvironment [11–14]. These studies demonstrated an enhanced permeability and retention (EPR) effect, via selective targeting that enables the accumulation of therapeutic agents in tumor tissues.

In the last several years, research on lipoprotein-based drug delivery carriers has shown a dramatic increase [15–19]. Our laboratory and others have shown have emphasized the favorable properties of synthetic/reconstituted HDL and HDL mimicking nanomaterials due to their biocompatibility, small size, nonimmunogenicity, long circulation time, and selective uptake via SR-B1 receptors [20–25]. The rHDL NPs also seem suitable to cross the blood brain barrier (BBB) [21] and to subsequently deliver their therapeutic payload to the brain. Fung et al. [26] demonstrated that HDL is able to cross the BBB by transcytosis, a unique mechanism facilitated by the scavenger receptor B-1 (SR-B1). Others have described the ability of a major HDL component, Apolipoprotein A-I (Apo A-I), to cross the BBB [27–29]. Kadiyala et al. [30] studied the ability of synthetic HDL (sHDL) nanodiscs as a chemoimmunotherapy for delivery of CpG, a Toll-like receptor 9 (TLR9) agonist, together with docetaxel (DTX), to the GBM microenvironment and elicit tumor regression. This combination of DTX-sHDL-CpG treatment with radiation (IR) resulted in tumor regression and long-term survival in 80% of GBM-bearing animals.

Our laboratory was one of the first groups to develop an rHDL drug delivery platform that mimics the properties of endogenous HDL, a cholesterol transport vehicle [31]. These rHDL NPs also seem suitable to cross the blood brain barrier (BBB) and subsequently deliver their therapeutic payload to the brain. The purpose of this study was to obtain* proof of concept data to support more advanced preclinical studies and to facilitate the eventual translation of these findings toward clinical applications*. To accomplish these goals, the drug Everolimus (EVR) was chosen due to the recent clinical interest in EVR and regarding the off-target effects associated with mammalian Target of Rapamycin (mTOR) inhibitors [32]. EVR is an FDA approved mTOR inhibitor that has been used in combination with temozolomide (TMZ) in a recent Phase II clinical study with GBM patients [33].

With a partition coefficient (X_log_P) value of 5.9, EVR is a suitable candidate to accumulate in the core regions of the rHDL “magic bullet” drug carrier [34]. The preferred payload for rHDL NPs appears to be hydrophobic compounds as their natural counterparts (HDL) transport primarily highly lipophilic cholesteryl esters as their core components [35]. The tumor selectivity of this drug delivery system is based on the overexpression of the SR-B1 receptor by cancer cells and tumors [36], including aggressive GBM cell lines. This feature provides enhanced targeting via the HDL type NPs for the SR-B1 receptor, thus limiting the off-target effects of chemotherapy [37].

## 2. Materials and Methods

Materials: Apolipoprotein A-I (Apo A-I) was purchased from MC Labs, San Francisco, CA. EVR and TMZ were purchased from Selleckchem, Houston, TX. Egg yolk phosphatidylcholine, free cholesterol and cholesterol oleate, and dimethyl-sulfoxide were obtained from Sigma Aldrich, St. Louis, MO.

### 2.1. Methods

#### 2.1.1. Preparation of the rHDL/EVR Nanoparticles

rHDL/EVR nanoparticles were assembled via a modified cholate dialysis procedure [38] as follows. Briefly, the lipid ingredients, 15 mg egg yolk PC (EYPC), 0.035 mg free cholesterol (FC), and 0.15 mg cholesteryl oleate (CE), were dissolved in chloroform and combined with the drug (EVR). The mixture of the lipids (PC, FC, and CE) and the drug (EVE) were dried under N_2_ to a thin film and dispersed in 60 *μ*L dimethyl sulphoxide (DMSO). To this mixture, 5 mg of Apo A-I and 140 *μ*L of 0.1 M sodium cholate were added and the volume was made up to 2 mL with Tris-EDTA buffer (10 mM Tris, 0.1 M KCl, and 1 mM EDTA pH 8.0). The final molar ratio of Apo A1: EYPC:FC:CE = 1:110:0.5:1.3. This procedure was optimized for EVR via a thermocycling/sonication step as follows. The EVR formulation was incubated at 50°C for 2 minutes and kept on ice for another 2 minutes. The formulation was then sonicated for 5 minutes with an Ultrasonic Processor™ at amplitude 25. The thermocycling/sonication procedure was repeated and the formulation was subsequently incubated for 12 hours at 4°C on a nutator shaker. Next the sample was dialyzed against 2 L of PBS, for 48 hours, with three buffer changes. The preparation was then centrifuged at 1000 rpm for 2 minutes and sterilized by passing it through a 0.2 *μ*m syringe filter and kept in the dark at 4°C until used.

#### 2.1.2. Characterization of rHDL/EVR Nanoparticles

The assembled, drug containing NPs were characterized with regard to physical properties and chemical composition. A bicinchoninic acid (BCA) assay (Thermo Fischer Scientific, Waltham, MA) was used to determine the Apo A-I. The Cholesterol E and Phospholipid C reagent kits (Wako Chemicals USA, Inc., Richmond, VA, USA) were used to determine the amount of cholesterol, cholesterol oleate, and EYPC in the NPs. These assays were conducted on a Cytation 3 Imaging reader Instrument (Bio-Tek, Winooski, VT, USA). The amount of EVR incorporated into the NPs was estimated by spectrophotometry at 277 nm (at the *ƛ*_max_ of EVR). To account for the interference by the Apo A1 protein (known to also absorb in this wavelength range), the absorbance of the empty rHDL NPs (containing the same amount of Apo A-I as the drug containing NPs) was subtracted from the rHDL/EVR absorbance.

The drug entrapment efficiency (DEE) was calculated using the equation

(1) $$\begin{array}{c} DEE=\left[\;\frac{\left(drug\;\;incorporated\;\;into\;\;NPs\right)}{\left(initial\;\;drug\right)}\;\right]\times 100\%. \end{array}$$

The percentage of individual components is calculated by a formula

(2) %  Component=Average  mg/mL  of  the  component∑  total  mg/mL  of  all  the  components×100

#### 2.1.3. Nanoparticle Size Measurements

To determine the diameter of the rHDL NPs, dynamic light scattering (DLS) was carried out using a DelsaNano HC Particle Analyzer (Beckman Coulter, Inc., Fullerton, CA). The data are reported as mean diameter ^±^ SD and poly-dispersity index (PDI) using number distribution analysis.

#### 2.1.4. Transmission Electron Microscopy

The rHDL NP solution was dialyzed against distilled water for 18-24hr. Carbon coated 200 mesh Formvar grids were used for sample preparation. Diluted samples were drop coated on to discharged grids, followed by staining with 1% uranyl acetate for 1 min. TEM images were taken using TecnaiTM Spirit electron microscope (EMCF facility in University of Texas Southwestern Medical Center, Dallas, TX).

#### 2.1.5. Cell Culture

A172, LN229, and LN18 cells were purchased from ATCC, Manassas, VA. Astrocytes and U87-MG were provided by Dr. Meharvan Singh (UNTHSC). Cells were cultured in DMEM, supplemented with 10% fetal bovine serum (FBS) and 1% (100 U/mL) Penicillin-Streptomycin. All cultures were incubated at 37°C in 5% CO_2_ in 75 cm^2^ flasks and passaged with 0.25% trypsin upon 80-90% confluency.

#### 2.1.6. Western Blot and Densitometry

Antibodies for SR-B1 and beta actin were acquired from Abcam (Cambridge, MA). Anti-rabbit secondary antibody was purchased from Cell Signaling Technology, Inc. (Beverly, MA). Cells were first pelleted and then disrupted using a lysis buffer (50 mM Tris-HCl (pH 8.0), 150 mM NaCl, 0.02% Sodium Azide, 100 *μ*g/ml PMSF, 1 *μ*g/ml aprotinin, 1% NP-4D). The protein concentration of each sample was determined using a BCA assay. A sample containing 20 *μ*g of protein (from each cell extract) was subjected to SDS electrophoresis. The isolated protein band was then transferred to a nitrocellulose membrane using the iBlot 2 system (Thermo Fisher Scientific, Carlsbad, CA) and probed with primary and secondary antibodies. Finally, the immune-complexes were visualized via chemiluminescence and were quantified using Image J software to yield the relative expression levels of SR-B1/Actin.

#### 2.1.7. Cytotoxicity Assays and IC_50_ Determination

Cell lines were grown in DMEM medium and incubated at 37°C with 5% CO_2_. Upon achieving the needed density, the cells were recovered, treated with trypan blue, and subsequently counted with a Cellometer mini (Nexcelom Bioscience, Lawrence, MA, USA). The cells were next seeded at a density of 5 × 10^4^ cells/well in 96 well microtiter plates in DMEM+10%FBS+1% penicillin/streptomycin medium and incubated for 24 hours. Once the cells were attached, the medium was removed, and the cells were washed with sterile PBS. Each cell line was subsequently incubated with increasing amounts of the respective drug formulations in DMEM+1% FBS for 72 hours. The drug containing NPs was prepared fresh for each experiment and stored at −20°C if necessary. Drug formulations were diluted in DMEM+1% FBS on the day of the experiment to 0.1-100 *μ*M. After incubation, 10 *μ*L of tetrazolium salt solution from the Cell Counting Kit-8 (CCK-8, Dojindo Molecular Technologies, Inc. Gaithersburg MD) was added to the respective wells and incubated for 3 hours. The absorbance (for each well) at 450 nm was obtained on a microplate reader (PowerWave, Biotek Instruments Inc., Winooski, VT, USA). IC_50_ values were calculated by plotting cell viability vs. drug concentration, where the molar concentration of the drug inducing a 50% reduction in viability was designated as the IC_50_ value for each formulation.

#### 2.1.8. Matrigel Invasion Assay

Corning BioCoat Matrigel Invasion Chambers (Corning, Bedford, MA) were used to assess the invasive limiting capabilities of the rHDL NPs, according to manufacturer's protocol. Typically, 25,000 cells/insert (0.5 ml) were placed onto rehydrated matrigel invasion chambers, which were then placed into a 24-well plate containing 0.75 ml of a chemoattractant (FBS). The cells were treated, respectively, with 5 *μ*M rHDL/EVR or free EVR (control). The cell suspensions were then incubated for 22 hours (37°C, 5% CO_2_). After incubation, noninvading cells were removed from the upper surface of the membrane by scrubbing with a cotton swab. The chambers were then removed from the wells, containing the chemoattractant, dipped in wells containing PBS for a quick wash, and immediately fixed with 100% methanol for 10 minutes. After drying the preparations were stained with crystal violet for 10 minutes. The membranes inside the chambers were then removed with a scalpel and placed onto an oil immersion slide to be viewed for invasion quantification. Fields in triplicate were randomly chosen for each treatment, and cells that invaded through the membrane were counted to assess the invasion.

#### 2.1.9. Cell Cycle Analysis

The respective cell lines were seeded (800,000 cells/dish) in 60 mm dishes and grown to confluency in DMEM+10% FBS+1% penicillin/streptomycin medium. After 24 hours, the medium was removed, and the cells were washed 3 times with PBS, pH 7.4. The cells were then incubated in serum free DMEM medium with free drug, rHDL/EVR, or empty HDL, with a positive control (no drug treatment). A control experiment was run by adding the same amount of DMSO as used to dissolve the free drug. After 22 hours, the medium of each preparation was removed, and the cells were washed twice with PBS. These suspensions were isolated and added to the corresponding samples of medium to ensure that all cells were covered. The cells were then harvested using trypsin as described above, pelleted, and resuspended in PBS+0.1%, bovine serum albumin (BSA).

To this suspension, 3 ml of cold absolute ethanol was added dropwise while vortexing. The cells were then fixed at 4°C overnight and then were again centrifuged and washed with PBS. Next, propidium iodide staining solution (Sigma, P4170) was mixed with the cell pellet. RNaseA stock solution (Worthington Biochemical, LS005649) was then added to each suspension, and the respective preparations were incubated at 37°C for 30 minutes. Cell cycle analysis was then performed with an FC500 Cytometer, with the data presented under Results.

#### 2.1.10. Stability Study

Aliquots of the rHDL/EVR NP formulations were placed into plastic vials and stored at either -20 or 4°C for 1, 15, and 30 days. At each time point, these aliquots were dialyzed as before and the percentage drug retention was determined.

## 3. Results

### 3.1. Physicochemical Properties of rHDL/EVR NPs

The chemical composition of the rHDL/EVR nanoparticles is shown in Table 1. The EVR loading efficiency into the rHDL NPs was similar to formulations described earlier [22]. The entrapment efficiency (EE) of EVR into the rHDL NPs was 57±5.6% while the most abundant component was phospholipid (60.7%) followed by protein (20.1%), cholesterol 10%, and EVR (9.3%).

**Figure 1:**
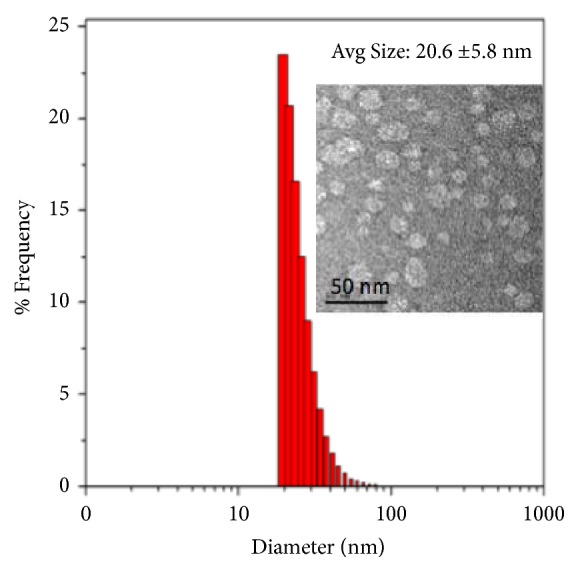
Estimation of rHDL/EVR nanoparticle size via dynamic light scattering (DLS) and Transmission electron microscopy (TEM).

The particle diameter analyses obtained from DLS measurements for the rHDL/ EVR NPs (Figure 1) show a mean diameter of 20.6 nm ± 5.8 nm. The polydispersity index (PDI) of the formulation was 0.253. The morphology and size of the particles were confirmed via transmission electron microscopy (see inset in Figure 1) where most of the NPs were observed to be smaller than 40 nm.

### 3.2. Storage Stability

The storage stability of the rHDL/EVR NPs was tested at two different temperatures 4°C and −20°C at 3 different time points (Table 2). The % drug recovery is calculated as % encapsulation efficiency (EE). At 4°C, the drug recovery dropped from 99% to 91% over a month. At −20°C almost 96% of the drug was recovered after one month (Table 2).

### 3.3. SR-B1 Expression in GBM Cells

**Figure 2:**
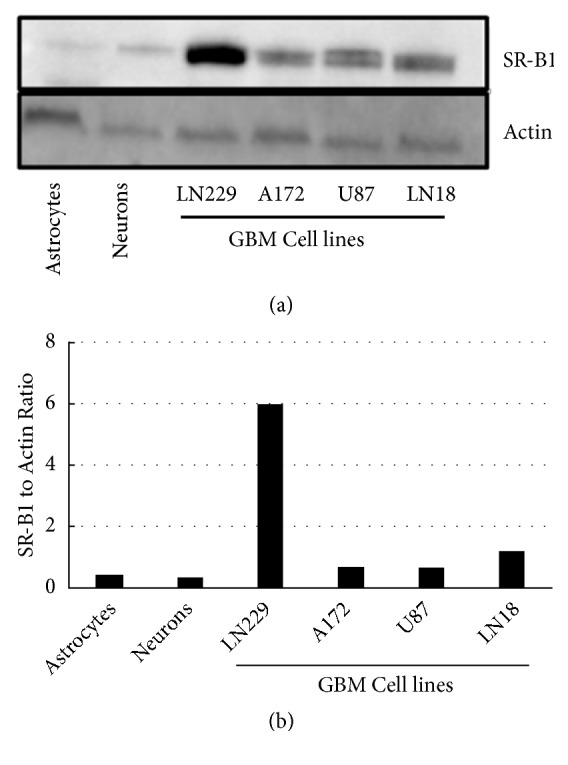
SR-B1 expression in GBM and nonmalignant cell lines via (a) Western blot and (b) densitometry.

A panel of GBM cells as well as primary astrocytes was probed for SR-B1 expression (Figure 2(a)) via a Western blot and densitometric analysis (Figure 2(b)). The LN229 cells, derived from an epithelial cell line, were found to have a relatively high SR-B1 expression, while U87-MG, a cerebellar cell line, was found to have relatively low SR-B1 expression. These two cell lines were chosen for subsequent cytotoxicity studies to determine their respective sensitivity to the rHDL/EVR formulation.

### 3.4. SR-B1 Expression as a Predictor of Sensitivity to rHDL/EVR NPs

Cell viability assays revealed the relative sensitivity of LN229, U87-MG, and astrocytes to rHDL/EVR as 1:3.4:55.9, respectively. These data indicate that the sensitivity of GBM cell lines to the drug containing rHDL NPs may be dependent on their respective SR-B1 expression and that the IC_50_ of EVR was substantially decreased against GBM cells, when delivered in rHDL NPs. The rHDL/EVR NPs also outperformed the free TMZ (the IC_50_ of TMZ was greater than 50 *μ*M against both GBM cell lines {data  not  shown}). In addition, treatment with empty rHDL NPs had a modest cytotoxic effect against GBM cells, perhaps due to the interaction of Apo A1 with the rHDL receptor [23].

**Figure 3:**
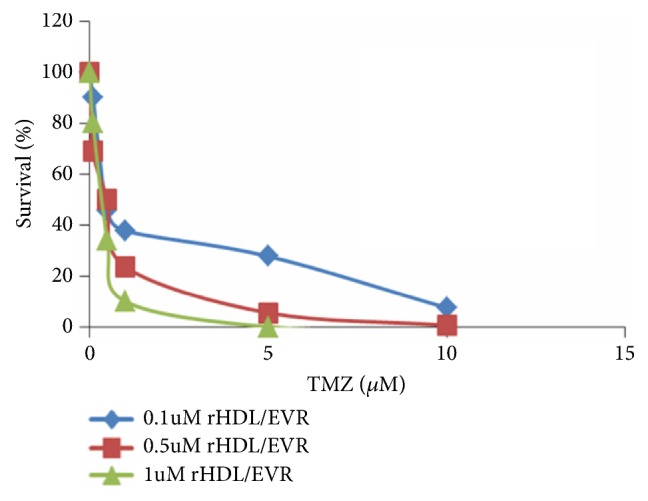
Survival of the LN229 cell line against rHDL EVR+TMZ combination treatment.

As shown in Table 3, the rHDL/EVR nanoparticles markedly potentiated the cytotoxic effect of EVR. Subsequently, a combination of EVR and TMZ, against the highest SR-B1-expressing GBM line (LN229), was also assessed. As seen in Figure 3, free TMZ further enhanced the impact of rHDL/EVR on the GMB (LN229) cells.

### 3.5. Estimation of Migration/ Invasion Tendencies of GBM Cells

**Figure 4:**
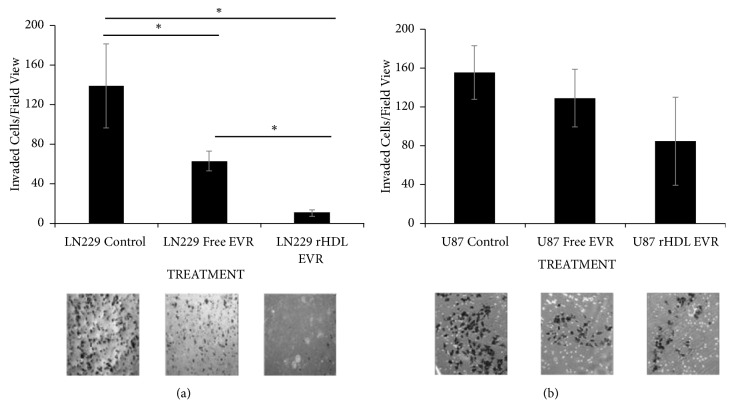
Comparison of the tendency for migration/invasion between GBM cell lines LN229 (a) and U87 (b) with differing SR-B1 expression. *∗*Statistically significant (p<0.05). No significant difference was found among different groups for U87 cell line.

The GBM cell lines, U87-MG (low SR-B1 expressor) and LN229 (a high SR-B1 expressor), were subjected to a matrigel invasion assay in the presence of either free EVR or rHDL/EVR. During rHDL/EVR treatment, invasion was almost completely eliminated in the high SR-B1 expressing cell line (LN229, Figure 4). In addition, the suppression of the migration/invasion tendencies by rHDL/EVR between the low expressor GBM cell lines (U87) and the high expressor cell line (LN229) was significantly different (*∗*p<0.05). The tendencies for migration/invasion of the LN229 cells were 13.87 times lower when treated with rHDL/EVR compared to 2.22 times lower tendencies when treated with free EVR (Figure 4) and as such indicate lower propensity for metastatic growth.

### 3.6. Cell Cycle Analysis of GBM Cells in Response to rHDL/EVR NPs via Flow Cytometry

**Figure 5:**
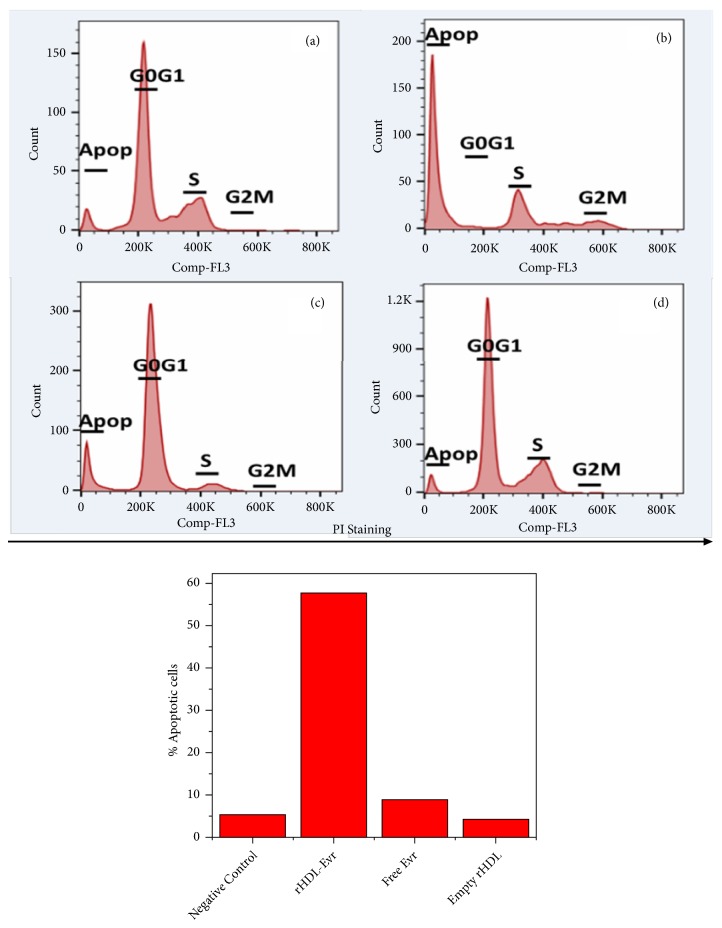
Cell cycle analysis of (a) LN229, negative control, (b) LN229 treated with rHDL/EVR, (c) LN229 treated with Free EVR, and (d) LN229 treated with empty rHDL.

Next we performed cell cycle analyses to assess the response of GBM cells to EVR and rHDL/EVR with regard to their tendencies to become apoptotic. As shown in Figure 5, treatment with 0.27 uM dose equivalent EVR in rHDL formulation resulted in a marked increase in apoptosis (not expected for a cytostatic agent) while the cell cycle was observed to be arrested at S phase, with less than 1% of the population reaching the G2/M phase, following treatment. Treatment with rHDL/EVR resulted in a 57.7% increase in the apoptotic population of LN229 cells (5.8 times higher than apoptotic population in LN 229 cells treated with EVR). rHDL/EVR also induced 4.3 times more apoptosis in LN229 cells than in U87-MG cells (data not shown). These apoptotic tendencies also correlate well with their respective SR-B1 expression (4.88:1 in favor of the LN229 cells vs. U87-MG cells). To control for the possibility of rHDL being cytotoxic itself, LN229 cells were subjected to empty rHDL treatment. These studies resulted in only 9% of the cell population being apoptotic following treatment (Figure 5).

## 4. Discussion

Traditional chemotherapy approaches generally produce off-target effects, including toxicity to normal tissues. The rHDL NPs are ideal candidates to protect against the off-target toxicity of drugs, as their hydrophobic core shields the therapeutic agent from release to nonmalignant tissues [6, 24].

Cholesterol transport is a highly conserved and regulated process in the CNS. While only a fraction of the lipoproteins found in the CNS is synthesized* in situ *by astrocytes and oligodendrocytes, most cranial lipoproteins and their components, including Apo A-I, originate from the blood and subsequently cross into the BBB [39]. The SR-B1 receptor is expressed in both astrocytes and neurons, perhaps facilitating the receptor-mediated transport of cholesterol. The SR-B1 expression, however, is far lower than the expression of low density lipoprotein receptor (LDL-R), the predominant receptor for Apolipoprotein E (ApoE) containing lipoproteins [40, 41]. Due to its limited expression in normal compared to much higher levels of expression seen in GBM cells (Figure 2) the SR-B1 receptor is thus a potential novel target for rHDL facilitated therapeutics that could mitigate the off-target cytotoxicity seen in traditional approaches to treat GBM.

**Figure 6:**
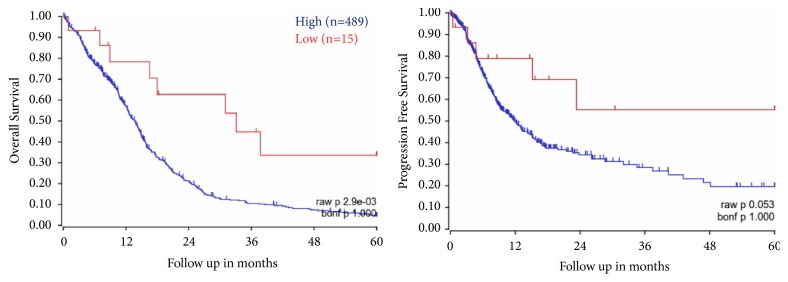
Overall and progression free survival of GBM patients as function of SCARB1 mRNA expression. Curves generated using R2 Genomics Platform and TCGA datasets (acquired from: https://hgserver1.amc.nl/cgi-bin/r2/main.cgi).

We have also evaluated the SR-B1 expression in GBM and survival of patients from an existing TCGA dataset. Kaplan-Meier survival curves for SCARB1 were generated by using R2 genomics and visualization platform. Database (Tumor Glioblastoma-TCGA-540) with survival information was chosen for analysis. The “Kaplan scan” of R2 genomics generates a Kaplan-Meier Plot based on the most optimal mRNA cut-off expression levels to discriminate between a good and bad prognosis cohort. Five-year survival was analyzed and plotted with event-free and overall survival based on survivin expression. It is evident that high SCARB1 expression in GBM correlates well with worse outcome (Figure 6).

The physicochemical characterization of the rHDL/EVR NPs reveal that these particles had similar properties to those rHDL formulations reported earlier in the literature [38, 42]. The EVR NPs examined in this study, contained 9.3% of the drug with the incorporation efficiency (EE) of about 60%. The small size of these NPs (~20 nm in diameter; Figure 1) should allow them to penetrate the interfibrillar domains of tumors, resulting in greater therapeutic efficacy and accumulation of drugs in the tumor mass [43]. The long circulation time of 3-5 days and small size [15, 35, 43] are anticipated to provide advantages for the rHDL “Trojan Horse” drug delivery system [34] over liposomal and other nanocarriers [44].

The cytotoxicity studies (Table 3) indicated that rHDL/EVR formulation was 185 times more potent than free EVR against LN 229 cells, whereas the free EVR was 3.2 times more effective against U87 cells. This discrepancy is likely to be due to the difference in the expression if the SR-B1 receptors (much higher in LN 229 cells compared to U87 cells).

This study highlights the capability of the rHDL NPs to deliver a targeted payload with multimodal mechanisms of action against GBM. It also provides proof of concept regarding the efficacy of delivering a hydrophobic; FDA approved mTOR inhibitor by utilizing transport system targeted to the SR-B1 receptor that is upregulated in most cancers, including GBM.

## Figures and Tables

**Table 1:** Chemical composition of rHDL/EVR nanoparticles.

Component	mg/mL ± SD	Component % of total ± SD
EVR	0.57 ± 0.09	9.3 ± 1.4
Protein	1.23 ± 0.04	20.1 ± 0.7
Phospholipid	3.72 ± 0.19	60.7 ± 3.2
Cholesterol	0.61 ± 0.04	10.0 ± 0.6

**Table 2:** Storage stability of the rHDL/EVR nanoparticles at different storage conditions.

STORAGE	STORAGE duration (days)			
Temperature	0	1	15	30
%EE*∗*at 4°C	100±4	99±5	96±7	91±3
% EE at -20°C	100±6	99±3	95±6	96±5

Note: *∗*EE is percent encapsulation efficiency.

**Table 3:** IC_50_ of everolimus (EVR) formulations against glioblastoma cells (LN 229 and U87) and astrocytes.

Cell line	Free-EVR (*µ*M)	rHDL-EVR (*µ*M)
LN229	>50	0.27±0.05
U87	2.91±0.3	0.92±0.17
Astrocytes	4.27±0.82	15.1±1.24

Note: a range of concentration of 10 nM to 50 *µ*M of each formulation was used for the cytotoxicity studies.

## Data Availability

The data presented in this paper is available and open for all readers and interested parties upon request from the corresponding author.

## Abbreviations

CNS: Central nervous system
EYPC: Egg yolk phosphatidylcholine
EVR: Everolimus
GBM: Glioblastoma
HDL: High density lipoprotein
mTOR: Mammalian Target of Rapamycin
NP: Nanoparticle
SR-B1: Scavenger receptor B type 1
TMZ: Temozolomide
BBB: Blood brain barrier
CNS: Central nervous system
EE: Entrapment efficiency
PDI: Polydispersity index
HIF-1a: Hypoxia inducible factor alpha.
